# Profile and potential role of novel metabolite biomarkers, especially indoleacrylic acid, in pathogenesis of neuromyelitis optica spectrum disorders

**DOI:** 10.3389/fphar.2023.1166085

**Published:** 2023-05-19

**Authors:** Jiangping Bian, Jiali Sun, Haoxiao Chang, Yuzhen Wei, Hengri Cong, Mengyuan Yao, Fuyao Xiao, Huabing Wang, Yaobo Zhao, Jianghong Liu, Xinghu Zhang, Linlin Yin

**Affiliations:** ^1^ Department of Neuroinfection and Neuroimmunology, Beijing Tiantan Hospital, Capital Medical University, Beijing, China; ^2^ China National Clinical Research Center for Neurological Diseases, Beijing, China; ^3^ Department of Neurology, Xuanwu Hospital, Capital Medical University, Beijing, China; ^4^ Beijing Institute of Brain Disorders, Collaborative Innovation Center for Brain Disorders, Capital Medical University, Beijing, China

**Keywords:** neuromyelitis optica spectrum disorders, aromatic amino acids and metabolites, liquid chromatography mass spectrometry, humoral fluids, indoleacrylic acid

## Abstract

**Background:** Neuromyelitis optica spectrum disorder (NMOSD) is an autoimmune central nervous system (CNS) inflammatory and demyelinating disorder that can lead to serious disability and mortality. Humoral fluid biomarkers with specific, convenient, and efficient profiles that could characterize and monitor disease activity or severity are very useful. We aimed to develop a sensitive and high-throughput liquid chromatography–tandem mass spectrometry (LC-MS)/MS-based analytical method for novel biomarkers finding in NMOSD patients and verified its function tentatively.

**Methods:** Serum samples were collected from 47 NMOSD patients, 18 patients with other neurological disorders (ONDs), and 35 healthy controls (HC). Cerebrospinal fluid (CSF) samples were collected from 18 NMOSD and 17 OND patients. Three aromatic amino acids (phenylalanine, tyrosine, and tryptophan) and nine important metabolites that included phenylacetylglutamine (PAGln), indoleacrylic acid (IA), 3-indole acetic acid (IAA), 5-hydroxyindoleacetic acid (HIAA), hippuric acid (HA), I-3-carboxylic acid (I-3-CA), kynurenine (KYN), kynurenic acid (KYNA), and quinine (QUIN) were analyzed by using the liquid chromatography–tandem mass spectrometry (LC-MS/MS)-based method. The profile of IA was further analyzed, and its function was verified in an astrocyte injury model stimulated by NMO-IgG, which represents important events in NMOSD pathogenesis.

**Results:** In the serum, tyrosine and some of the tryptophan metabolites IA and I-3-CA decreased, and HIAA increased significantly in NMOSD patients. The CSF levels of phenylalanine and tyrosine showed a significant increase exactly during the relapse stage, and IA in the CSF was also increased markedly during the relapse and remission phases. All conversion ratios had similar profiles with their level fluctuations. In addition, the serum IA levels negatively correlated with glial fibrillary acidic protein (GFAP), and neurofilament light (NfL) levels in the serum of NMOSD patients were measured by using ultra-sensitive single-molecule arrays (Simoa). IA showed an anti-inflammatory effect in an *in vitro* astrocyte injury model.

**Conclusion:** Our data suggest that essential aromatic amino acid tryptophan metabolites IA in the serum or CSF may serve as a novel promising biomarker to monitor and predict the activity and severity of NMOSD disease. Supplying or enhancing IA function can promote anti-inflammatory responses and may have therapeutic benefits.

## Introduction

Neuromyelitis optica spectrum disorder (NMOSD) is an autoimmune disease associated with central nervous system (CNS) inflammation and demyelination and is characterized by a frequent relapsing course that mainly affects the optic nerves and spinal cord, resulting in optic neuritis, transverse myelitis, and often severe disability (Wingerchuk et al., 2015). Although NMOSD is rare, it occurs all over the world and is especially more prevalent in non-Caucasians and observed in Asian countries (Hor et al., 2020), with a high incidence in women aged from 45 to 65 years. The incidence of NMOSD in China per 100,000 person-years is 0.278, with 0.347 in adults (Tian et al., 2020).

The pathology of NMOSD is complicated, with recurrent attacks of the target antigen aquaporin-4 water channel enriched on the foot processes of astrocytes by a highly specific serum autoantibody (NMO-IgG) being confirmed as one of the primary pathogenesis (Wingerchuk et al., 2015; Huda et al., 2019). Patients often develop severe residual disability from repeated attacks; therefore, early diagnosis, accurate prediction of relapse, and initiation of attack-preventing medications are very important to improve the prognosis. miRNAs or hyperphosphorylation of specific signaling pathways have been implicated in neurological dysfunction diseases (Jiang et al., 2019; Zeng et al., 2022). To identify more specific, convenient, and efficient biomarkers, a novel approach with the ability to characterize and monitor disease activity and severity in patients with NMOSD should be developed urgently.

In recent years, advanced metabolite profiling of body fluids like serum/plasma, cerebrospinal fluid (CSF), or urine, known as “metabolomics,” has become a powerful and promising tool to identify novel biomarkers or “metabolic fingerprints” characteristic for various diseases at different stages (Arneth et al., 2019; Fitzgerald et al., 2021). According to our knowledge, no publications have been reported so far that have described the profile of aromatic amino acids and their metabolites in the serum or CSF of NMOSD patients. Metabolic abnormalities have been observed on MR spectroscopy in NMO patients of normal-appearing white matter (NAWM) and normal-appearing gray matter (NAGM) when compared with healthy subjects (de Seze et al., 2010). They found that N-acetyl-aspartate (NAA) was often decreased, and choline was found increased in NMO patients, even in NAWM. As we know, microbes (*Peptostreptococcus* species) metabolize tryptophan (Trp) to IA by utilizing intestinal mucins (Wlodarska et al., 2017). Although how microbiota dysbiosis contributes to the onset and progression of NMOSD remains unclear and requires further study, reports reveal dysbiosis of intestinal bacteria in patients with NMOSD (Shi et al., 2020). Therefore, we aimed to develop a sensitive and high-throughput liquid chromatography–tandem mass spectrometry (LC-MS)/MS-based analytical method for the simultaneous determination of three aromatic amino acids that includes phenylalanine (Phe), tyrosine, Trp, and their metabolites phenylacetylglutamine (PAGln), indoleacrylic acid (IA), 3-indole acetic acid (IAA), 5-hydroxyindoleacetic acid (HIAA), hippuric acid (HA), I-3-carboxylic acid (I-3-CA), kynurenine (KYN), kynurenic acid (KYNA), and quinine (QUIN) in the serum and CSF from NMOSD patients.

With the development of the ultra-sensitive single-molecule array (Simoa) approach, peripheral glial fibrillary acidic protein (GFAP) and neurofilament light (NfL) levels have been reported to indicate early astrocytic or neuronal axonal damage and as clinically useful biomarkers of disease activity and disability in NMOSD (Watanabe et al., 2019; Aktas et al., 2021; Chang et al., 2021; Chen et al., 2021; Schindler et al., 2021). As a result, associations between candidate biomarkers screening from aromatic amino acid metabolites and NfL or GFAP levels or clinical parameters in NMOSD patients have been assessed to evaluate technology sensitivity. Furthermore, the function of the novel candidate metabolic biomarker IA was measured in an *in vitro* damaged astrocytes model that represents one of the important events in NMOSD pathogenesis. Evaluation and characterization of a wide range of metabolic compositions in the serum and CSF would assist in understanding the basic pathogenesis, gaining knowledge about the pathophysiological process, and identifying biomarkers for the early diagnosis or prognosis of NMOSD.

## Materials and methods

### Chemical and reagents

Standards of Phe, tyrosine, and Trp and their metabolites PAGln, IA, IAA, HIAA, HA, I-3-CA, KYN, KYNA, and QUIN, as well as an internal standard (IS) were all purchased from Sigma-Aldrich (St. Louis, MO, United States). The reagents and chemicals utilized in this investigation were of analytical or LC-MS grade obtained from commercial sources. Water was deionized and filtered using a Milli-Q Plus apparatus (Millipore Corporation, Bedford, MA, United States). All standard stocks were stored at −80°C in light-protected containers in brown Eppendorf tubes.

### Sample collection

This study was approved by the Ethics Committee of Beijing Tiantan Hospital, Capital Medical University (KY 2021-069-01), and all participants gave their written informed permission. All serum and CSF samples from NMOSD and other neurological disorder (OND) patients were obtained before therapeutic administration. NMOSD was diagnosed based on 2015 Revised International Criteria (Wingerchuk et al., 2015). In serum collection, the OND group included patients with benign intracranial hypertension (n = 8), occasional limb numbness (n = 1), hypotensive headache (n = 1), venous sinus thrombosis (n = 1), psychogenic movement disorders (n = 1), normal pressure hydrocephalus (n = 1), Paget’s disease of bone (n = 1), benign paroxysmal positional vertigo (n = 1), sleep disturbance (n = 1), vitamin B12 deficiency (n = 1), and hypertension (n = 1). In the CSF collection, the OND group included patients with benign intracranial hypertension (n = 12), psychogenic movement disorders (n = 1), benign paroxysmal positional vertigo (n = 1), and multiple cranial nerve impairments (n = 3). None of the OND patients had any other autoimmune diseases.

### Serum

The serum samples were collected from 47 NMOSD patients at the Department of Neuroinfection and Neuroimmunology; 18 OND patients at the Department of Neurology, Beijing Tiantan Hospital affiliated with Capital Medical University, between May 2017 and September 2019; and 35 healthy controls (HC) from the Health Management Center, Beijing Tiantan Hospital Capital Medical University. The serum samples were centrifuged, immediately aliquoted, and stored in 1.5 mL polypropylene tubes at −80 °C until analysis. The clinical and demographic characteristics of all participants who donated serum samples are summarized in Table 1.

**TABLE 1:** Clinical and demographic information of participants who donated serum samples.

	NMOSD (n = 47)	NMOSD		HC (n = 35)	OND (n = 18)	*p*
		Relapse (n = 12)	Remission (n = 35)			
Gender (F/M)	42/5	11/1	31/4	14/21	10/8	<0.05^a^ ^,^ ^b^
Age, mean ± SD, years	37.9 ± 13.4	40.3 ± 12.3	37.0 ± 13.9	33.1 ± 11.0	43.2 ± 10.4	>0.05^c^
EDSS at measurement, median (range)	4.5 (1.0–8.5)	5.5 (3.5–8.5)	3.0 (1.0–8.5)	—	—	0.008^d^
Medication within last 6 months						
Glucocorticoids	25/47	9/12	16/35	—	—	
Gamma globulin	5/47	2/12	3/35	—	—	
Plasma exchange	2/47	0/12	2/35	—	—	
Disease-modifying therapy	21/47	4/12	17/35	—	—	
Neurotrophic drugs	7/47	4/12	3/35	—	—	
Traditional Chinese Medicine	3/47	3/12	0/35	—	—	

Continuous variables are shown as means ± SD, and noncontinuous variables are shown as the median (range). Count data are detected by the chi-square test.

NMOSD, neuromyelitis optica spectrum disorders; HC, healthy control; OND, other neurological diseases; EDSS, expanded disability status scale. Disease-modifying therapies include mycophenolate mofetil, rituximab, cyclophosphamide, and azathioprine.

^a^ NMOSD-remission vs*.* HC or OND.

^b^ MOSD-relapse vs*.* OND.

^c^ All detective groups compare with each other.

^d^ NMOSD-relapse vs*.* NMOSD-remission.

### Cerebrospinal fluid

The CSF samples from 18 NMOSD and 17 OND patients were collected at the Department of Neurology, Beijing Tiantan Hospital, between May 2017 and August 2019. Six precious CSF samples from NMOSD patients in their remission phase were collected. All CSF samples from the OND patients were collected before treatment during the acute stage. The CSF samples were centrifuged, immediately aliquoted, and stored in 1.5 mL polypropylene tubes at −80 °C until analysis. The clinical and demographic characteristics of all participants who donated the CSF samples are summarized in Table 2.

**TABLE 2:** Clinical and demographic information of participants who donated cerebrospinal fluid samples.

	NMOSD (n = 18)	NMOSD		OND (n = 17)	*p*
		Relapse (n = 12)	Remission (n = 6)		
Gender (F/M)	16/2	12/0	4/2	9/8	0.009^a^
Age, mean ± SD, years	40.1 ± 13.3	34.6 ± 9.6	51.2 ± 13.2	43.8 ± 10.7	0.008^b^
EDSS at measurement, median (range)	3.8 (1.0–8.0)	3.8 (2.0–8.0)	3.5 (1.0–5.5)	—	0.421^b^
Medication within last 6 months					
Glucocorticoids	14/18	10/12	4/6	—	
Gamma globulin	4/18	2/12	2/6	—	
Plasma exchange	1/18	1/12	0/6	—	
Disease-modifying therapy	9/18	5/12	4/6	—	
Neurotrophic drugs	13/18	8/12	5/6	—	
Traditional Chinese Medicine	1/18	0/12	1/6	—	

Continuous variables are shown as the means ± SD, and noncontinuous variables are shown as the median (range). Count data are detected by the chi-squared test.

NMOSD, neuromyelitis optica spectrum disorders; OND, other neurological diseases; EDSS, expanded disability status scale. Disease-modifying therapies include mycophenolate mofetil, rituximab, cyclophosphamide, and azathioprine.

^a^ NMOSD-relapse vs*.* OND.

^b^ NMOSD-relapse vs*.* NMOSD-remission.

### Sample preparation for liquid chromatography–tandem mass spectrometry chromatography

Before the experimental procedure, the samples were taken out of the refrigerator, thawed, and rewarmed to room temperature. Twenty microliters of serum or CSF samples with 80 µL of internal calibration standard was prepared according to a previous method (Fazio et al., 2015). Briefly, the serum samples were vortex-mixed for 30 s and centrifuged at 20,000×*g* for 20 min at 4 °C. Fifty microliters of the clean upper layer was transferred to a vial for the autosampler. After centrifugation at 3,000×*g*, 4 °C for 15 min, the supernatant of the CSF sample was collected and evaporated to dryness under a gentle stream of nitrogen. The dried extract was reconstituted to 40 µL of the acidified mobile phase. A total of 5 μL was injected into the LC-MS/MS chromatographic system.

### Liquid chromatography–tandem mass spectrometry system

The LC-MS/MS system consists of a SCIEX QTRAP 6500+ mass spectrometer (SCIEX, Foster City, CA, United States), coupled to HPLC binary pump and an autosampler (Shimadzu Scientific Instruments, INC., Columbia, MD, United States). The mass spectrometer was operated in the positive ESI mode with a capillary voltage of 5.5 kV. The temperature of desolvation was set at 550°C. Chromatographic separation was performed on a 5-µm EVO C18 column (2.1 × 100 mm, 2.6 μm, 100 Å pore size, 00F-4633-E0, Phenomenex Kinetex, United States). The column was maintained at 35°C. The mobile phase for LC analysis consisted of two solutions: A, methanol or isopropyl alcohol in water (3/2, v/v) combined with 0.2% formic acid (Merck, Darmstadt, Germany) and B, 5 mmol/L ammonium acetate. Elution was performed at a flow rate of 300 μL/min. The mobile phase gradient program started at 0%–40% of B (v/v) at 0–0.1 min, then 40%–100% of B (v/v) at 0.1–5.0 min, and held for 1.5 min at 100% of B. The column was equilibrated for 2–3 min. Data acquisition and processing were carried out using the Analyst^®^ software version 1.7.3 (SCIEX) and MultiQuant 3.2 (SCIEX). Chromatographic parameters for all detectable aromatic amino acids and metabolites are presented in Table 3. All sample measurements were done blinded, that is, the analysts were unaware of any clinical or diagnostic information.

**TABLE 3:** List of chromatographic parameters for all analytes.

Name	Molecular formula	RT (min)	MW (g/mol)	Q3 mass
Phe	C_9_H_11_NO_2_	3.20	166.1	120.1
Tyr	C_10_H_14_NO_3_	3.60	182.1	136.0
PAGln	C_13_H_11_D_5_N_2_O_4_	2.38	265.2	130.2
Trp	C_11_H_12_N_2_O_2_	1.86	205.1	146.0
IA	C_2_H_4_O_2_	1.83	188.1	115.0
HIAA	C_10_H_9_NO_2_	2.47	192.0	146.0
IAA	C_10_H_9_NO_2_	3.18	176.1	130.0
I-3-CA	C_9_H_7_NO_2_	1.84	146.1	118.1
HA	C_9_H_9_NO_3_	2.59	179.9	104.7

RT, retention time; MW, molecular weight; Phe, phenylalanine; Tyr, tyrosine; PAGln, phenylacetylglutamine; Trp, tryptophan; IA, indoleacrylic acid; HIAA, 5-hydroxyindole acetic acid; IAA, 3-indole acetic acid; I-3-CA, I-3-carboxylic acid; HA, hippuric acid.

In this study, ceramide labeled with a stable isotope (C24:1) was used as an IS. Linearity was determined using a series of putrescine solutions of 0, 1.25, 2.5, 5, 7.5, 10, 15, and 20 μM mixtures of 5 μM ceramide (C24:1). As shown previously, the calibration curve included a zero sample (only IS added). The calibration curves were constructed based on the peak area ratio of the ceramide to ceramide (C24:1).

### Measurement of NfL and GFAP in serum of NMOSD patients

Serum samples were centrifuged at 2,000×*g* for 10 min at room temperature and stored at −80°C within 3 h of collection. The serum levels of NfL and GFAP were measured using the Simoa technology by the ultra-high-sensitivity protein molecular detection instrument (Simoa HD-1, Quanterix, MA, United States) and Simoa NfL (502153, Quanterix, MA, United States) and GFAP (102336, Quanterix, MA, United States) reagent kits.

### Primary astrocyte culture and treatment

Primary astrocyte culture and NMO-IgG purification process have been described previously (Du L. S. et al., 2020; Wang et al., 2022). IgG was isolated from sterile-filtered serum pools (NMOSD with positive AQP4-IgG or HC) using HiTrap Protein G HP (GE Healthcare, Bio-sciences, Piscataway, NJ, United States). The serum samples were diluted 1:1 with a binding buffer. After filling the column with the binding buffer, the samples were applied to the columns. Thereafter, the columns were washed again with the binding buffer, and antibodies were eluted following the manufacturer’s directions. Finally, the antibodies were concentrated on Amicon Ultra-4 centrifugation units (Merck Millipore, Billerica, MA, United States) with 10,000 MW cut-offs. The titer of AQP4-IgG in NMOSD samples was 53.43 units/mL, determined by the AQP-4 Ab ELISA Kit (RSR LIMITED, Lot. 2KAQE62D). Finally, the concentrated IgG was sterile-filtered at 0.22 μm and then stored at −80 °C. Briefly, the mixed glial cultures were prepared from the cerebral cortices of 1-day postnatal rats. The mixed glial cells were filtered and cultured in Dulbecco’s modified Eagle’s medium/F12 (Gibco, Grand Island, NY, United States) supplemented with 10% heat-inactivated fetal bovine serum (Gibco) and 1% penicillin/streptomycin (Gibco) at 37 °C in a humidified atmosphere of 5% CO_2_-95% air. The medium was completely replaced every 3–4 days. At about day 12, microglia, endothelial cells, and oligodendrocyte lineage cells were removed from the mixed glial cells via shaking. More than 95% of the cells were positive for GFAP staining, which is a specific marker of astrocytes.

Astrocytes were treated with NMO-IgG (AQP4-IgG, 4 units/mL) at 37 °C for 4 h to mimic NMOSD damage. To determine the effects of IA or KYN on NMO-IgG-induced interleukin-6 (IL-6), chemokine (C-C motif) ligand 2 (CCL2), and aryl hydrocarbon receptor (AHR) expression, IA (40 µM) or KYN (50 µM) was pre-incubated with astrocytes before NMO-IgG stimulation.

### RNA isolation and quantitative-PCR

As described previously (Du L. et al., 2020; Wang et al., 2022), total RNA was isolated from the astrocytes using a TRIzol reagent (Invitrogen, Carlsbad, CA, United States). RNA purity and concentration were determined using a NanoDrop^®^ ND-1000 spectrophotometer (NanoDrop Technologies, Wilmington, DE, United States). RNA (1 µg) was subjected to cDNA synthesis by using the Roche Transcriptor First Strand cDNA Synthesis Kit (Roche, Basel, Switzerland) with anchored oligo (dT) and random hexamer primers. SYBR Green–based qPCR was performed to measure the relative mRNA expression of IL-6, CCL2, and AHR, which was normalized to that of GAPDH. All primers were designed and ordered from BGI Genomics Co., Ltd. The primer sequences are as follow: IL-6 *forward*, 5′-AGT​TGG​ATG​GTC​TTG​GTC​CTT​AGC-3′, IL-6 *reverse*, 5′-AGC​CAG​AGT​CAT​TCA​GAG​CAA​TAC​T-3′; CCL2 *forward*, 5′-AGG​TCT​CTG​TCA​CGC​TTC​TG-3′, CCL2 *reverse*, 5′-GTT​CTC​CAG​CCG​ACT​CAT​TG-3′; AHR *forward*, 5′-CTG​CTT​CAT​TTG​TCG​TGT​CC-3′, AHR *reverse*, 5′-TTT​CCT​TGG​AAC​TGC​ATA​GTC​A-3′; CYP1a1 *forward*, 5′-TTC​TCT​TTG​GTT​TGG​GCA​AG-3′, CYP1a1 *reverse*, 5′-GCC​CAT​AGG​CAG​GAG​TCA​TA-3′; CYP1b1 *forward*, 5′-GGA​CAA​GGA​CGG​CTT​CAT​TA-3′, CYP1b1 *reverse*, 5′-GCG​AGG​ATG​GAG​ATG​AAG​AG-3′; GAPDH *forward*, 5′-AAG​TTC​AAC​GGC​ACA​GTC​AAG-3′, GAPDH *reverse*, 5′-ACA​TAC​TCA​GCA​CCA​GCA​TCA-3′. The 2^−ΔΔCt^ method calculated the fold difference in expression relative to GAPDH.

### Statistical analysis

LC-MS raw data were processed using the Analyst 1.7.3 software (SCIEX) for peak integration. Metabolite peaks were compared against reference standards to confirm identities. All statistical analyses were performed using GraphPad Prism 8 (GraphPad Software, San Diego, CA, United States) and IBM SPSS Statistics. The significant differences between the groups were determined by a 2-tailed unpaired Student’s t-test or Mann–Whitney U test when the samples were not distributed normally. Count data were detected by the chi-squared test. All data are represented as mean ± standard deviation (SD). Each *in vitro* experiment was repeated at least thrice. The statistical significance level was assumed for *p* values < 0.05.

To investigate correlations between the metabolite levels in the serum or CSF and clinical parameter expanded disability status scale (EDSS), a two-tailed Spearman’s rank correlation coefficient was used to ascertain the associations. Univariate linear regression models and a 95% confidence interval were conducted. A value of *p* < 0.05 was considered statistically significant. Correlation analyses were performed only in the all enrolled NMOSD patients who provided serum or CSF samples and were not performed in the relapse/remission subgroups due to limited sample size.

## Results

### Demographics

The demographic and clinical characteristics of all subgroups (patients who provided serum samples) are presented in Table 1. Forty-seven patients with NMOSD (12 in relapse and 35 in remission), 18 patients with OND, and 35 healthy volunteers were recruited. There was no significant difference in the mean age among all groups (*p* > 0.05). We observed a significant difference in the gender distribution of NMOSD, where females accounted for a higher proportion, consistent with previous reports (Papp et al., 2021). More women in the NMOSD group during the remission (31/35) stage enrolled in this study than in the HC (14/21) and OND (10/8) groups (*p* = 0.013). Moreover, there were more women in the NMOSD group at the relapse stage (31/35) (*p* = 0.049) than in the OND groups (*p* = 0.049). The median EDSS score at the sample check-point of NMOSD during the relapse phase was 5.5 (3.5–8.5), which is significantly higher than for the NMOSD patients during the remission phase (3.0, 1.0–8.5, and *p* = 0.008). We also provide the medication information of enrolled patients within the last 6 months (Table 1). In addition, whether NMOSD patients obtained immunosuppressive drugs or not had no significant effect on the metabolite levels of the serum (Supplementary Figure S1).

Table 2 shows the demographic and clinical data of subjects who provided CSF samples. Twelve patients with NMOSD in relapse, 6 patients with NMOSD in remission, and 17 OND patients were recruited. There was a significant difference in the mean ages between the NMOSD patients in the relapse and remission groups (*p* = 0.008). A significantly higher proportion of female patients was observed in NMOSD patients of the relapse phase than in the OND group (*p* = 0.009). The median EDSS score at the time point of sample collection in the NMOSD patients during the relapse phase was 3.8 (2.0–8.0), and 3.5 (1.0–5.5) for NMOSD patients during the remission phase (*p =* 0.421). The medication information of the enrolled NMOSD patients within the last 6 months was provided (Table 2). Furthermore, whether NMOSD patients obtained immunosuppressive drugs or not had no significant effect the on metabolite levels of the CSF (Supplementary Figure S2).

### Aromatic amino acids and their metabolites in serum

A total of 12 aromatic amino acids and metabolite biomarkers were measured in the serum of NMOSD and OND patients or HC groups. However, the serum levels of KYNA, KYN, and QUIN were below the detectable thresholds due to sensitivity and technique limitations.

### Phe and its metabolites

**FIGURE 1:**
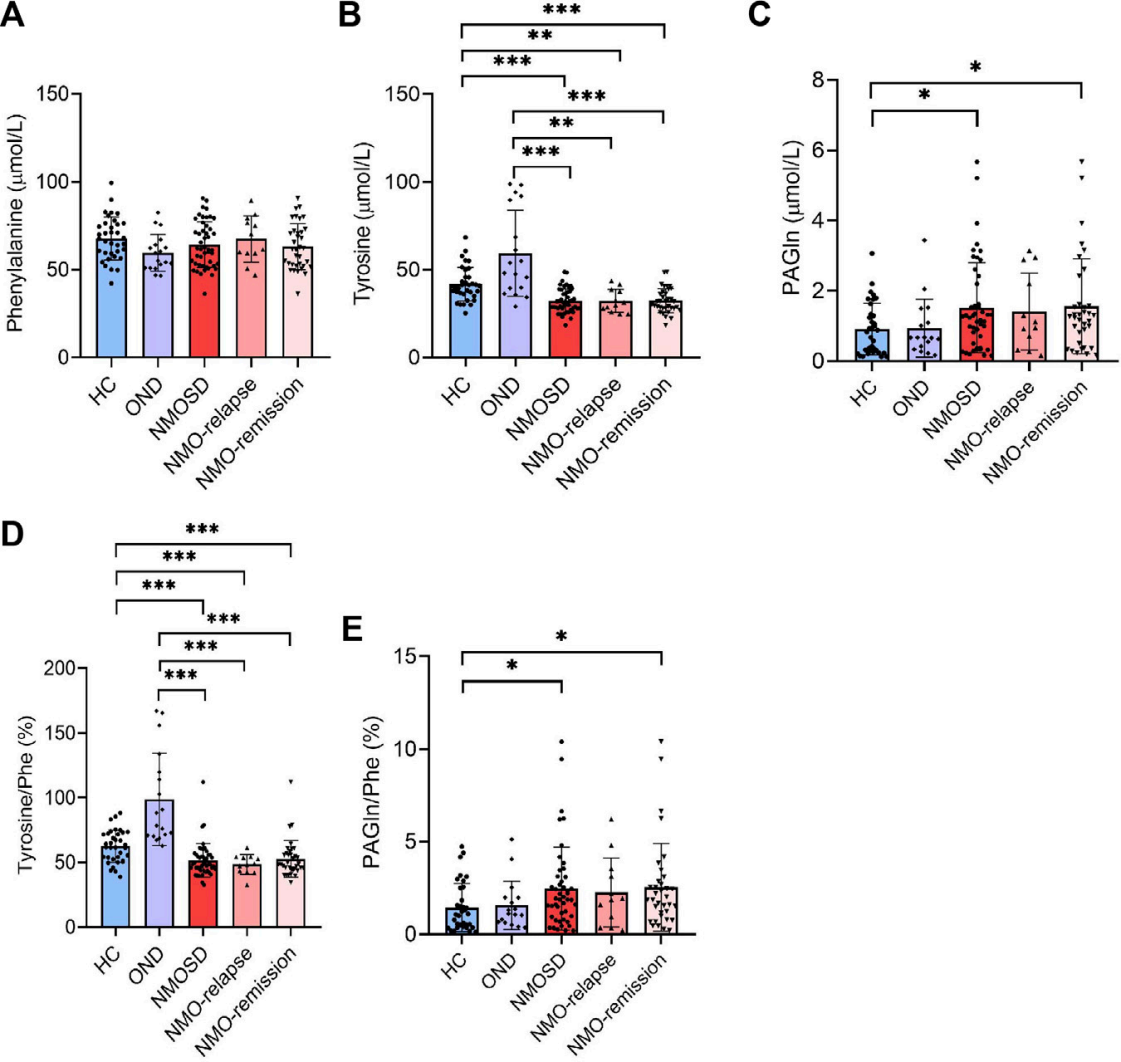
Levels of phenylalanine (Phe) and its metabolites in the peripheral blood of all participants and their conversion ratios. The serum of 35 healthy controls (HC), 18 with other neurological diseases (OND), and 47 (12 in relapse and 35 in remission) neuromyelitis optica spectrum disorder (NMOSD) patients were analyzed by liquid chromatography–tandem mass spectrometry (LC-MS/MS)-based method. Serum levels of Phe **(A)**, tyrosine **(B)**, and their metabolites phenylacetylglutamine [PAGln, **(C)**] were evaluated. Conversion ratio of tyrosine/Phe **(D)** and PAGln/Phe **(E)** among all detective groups. Statistical significance was determined by the Student’s t-test or Mann–Whitney U test. Data are presented as the mean ± SD. *p < 0.05, **p < 0.01, ***p < 0.001.

Phe, one of the aromatic amino acids, can convert into tyrosine by Phe hydroxylase. Figure 1 shows that serum concentrations of tyrosine decreased significantly in NMOSD patients at both relapse (32.3 ± 6.6 μmol/L) and remission (32.4 ± 7.0 μmol/L) phases, when they were compared with HC (41.8 ± 9.6 μmol/L and *p* = 0.003) or OND patients (59.3 ± 24.5 μmol/L and *p* < 0.001). However, there were no significant differences in the tyrosine levels between the two NMOSD subgroups (Figure 1B). The serum levels of PAGln were increased significantly in the NMOSD group (1.5 ± 1.3 μmol/L) and also in NMOSD patients during remission (1.6 ± 1.4 μmol/L) when compared to the HC group (0.9 ± 0.7 μmol/L and *p* < 0.05); no significant concentration alterations were found among all the other detective groups (Figure 1C). Furthermore, no significant difference was observed regarding Phe concentrations among all detective groups (Figure 1A).

Similarly, the conversion ratios of Phe into its metabolite tyrosine and PAGln were both analyzed. The ratio of tyrosine/Phe decreased markedly in NMOSD patients (51.7% ± 13.0%), both in the relapse (48.5% ± 7.6%) and remission (52.7% ± 14.3%) stages of NMOSD patients, when compared to those of the OND (98.7% ± 35.7%, *p* < 0.001) or HC (62.5% ± 12.4%, *p* < 0.001) group. No significant differences were found in the tyrosine/Phe ratio between the two NMOSD subgroups (Figure 1D). The conversion ratio of PAGln/Phe was significantly increased in the NMOSD group (2.5% ± 2.2%) and also in NMOSD patients during the remission phase (2.5% ± 2.4%) when compared to those in the HC group (1.5% ± 1.3%, *p* < 0.05); no significant conversion ratio was found among all the other detective groups (Figure 1E).

### Trp and its metabolites

**FIGURE 2:**
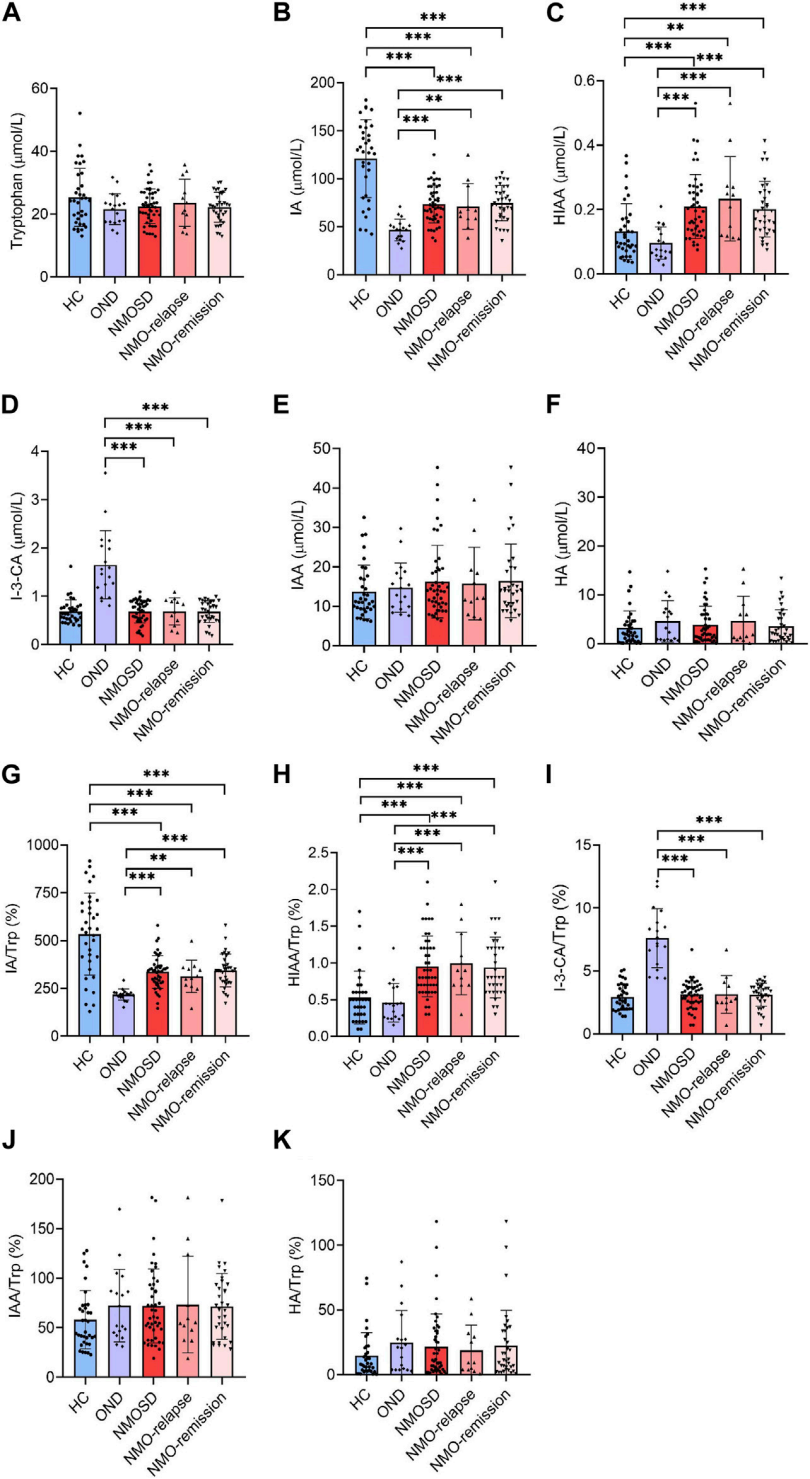
Serum levels of tryptophan (Trp) and its metabolites in all participants and their conversion ratios. All serum samples provided by subjects who included 35 healthy controls (HC), 18 with other neurological diseases (OND), and 47 (12 in relapse and 35 in remission) neuromyelitis optica spectrum disorder (NMOSD) patients were analyzed by liquid chromatography–tandem mass spectrometry (LC-MS/MS)-based method. Trp and its five metabolites of indole metabolism pathways were evaluated. Serum levels of Trp **(A)**, indoleacrylic acid [IA, **(B)**], 5-hydroxyindoleacetic acid [HIAA, **(C)**], I-3-carboxylic acid [I-3-CA, **(D)**], 3-indole acetic acid [IAA, **(E)**] and hippuric acid [HA, **(F)**] were evaluated. The conversion profiles of Trp metabolites IA/Trp **(G)**, HIAA/Trp **(H)**, I-3-CA/Trp **(I)**, IAA/Trp **(J)**, and HA/Trp **(K)** are shown. Statistical significance was determined by the Student’s t-test or Mann–Whitney U test; data are presented as the mean ± SD. **p < 0.01 and ***p < 0.001.

Trp is a nutritionally essential amino acid for both humans and animals. Trp and its metabolites are crucial for maintaining neurological function, immunity, and homeostasis in the body. In the present study, we measured Trp and its five metabolites in Trp metabolism pathways leading to serotonin and indole in the serum of NMOSD patients by using the specific LC-MS/MS method. Unexpectedly, no significant differences were found in the serum levels of Trp and its metabolites IAA and HA among all evaluated groups (Figures 2A,E,F). The serum levels of IA were markedly decreased in NMOSD patients (73.6 ± 19.6 μmol/L) when compared to the HC group (123.7 ± 43.5 μmol/L, *p* < 0.001) and also decreased during both the relapse (71.1 ± 23.7 μmol/L) and remission (74.5 ± 18.4 μmol/L) phases. Conversely, IA was markedly higher in NMOSD patients (73.6 ± 19.6 μmol/L) than in the OND group (46.6 ± 11.2 μmol/L and *p* < 0.001) and was also significantly increased during both the relapse and remission phases (*p* < 0.01 and *p* < 0.001, respectively) (Figure 2B). The serum concentrations of HIAA were significantly higher in NMOSD patients (0.2 ± 0.2 μmol/L) than in those of the HC (0.1 ± 0.1 μmol/L and *p* < 0.001) or OND (0.1 ± 0.1 μmol/L and *p* < 0.001) groups and also found increased obviously in the two subgroups of NMOSD patients (0.2 ± 0.1 and 0.2 ± 0.2 μmol/L for relapse and remission phase, respectively) (Figure 2C). Metabolite I-3-CA was significantly lower in NMOSD patients (0.7 ± 0.2 μmol/L) than in OND patients (1.6 ± 0.7 μmol/L, *p* < 0.001) and also found significantly decreased in both NMOSD subgroups (0.7 ± 0.3 and 0.7 ± 0.2 μmol/L for relapse and remission phases, respectively). No significant difference was found between the NMOSD and HC groups for serum levels of I-3-CA (Figure 2D). All Trp metabolites detected in this study showed no significant difference between the two subsets of NMOSD during the relapse and remission phases.

The conversion profiles of Trp metabolites IA/Trp, HIAA/Trp, I-3-CA/Trp, IAA/Trp, and HA/Trp showed similar tendencies to the level alteration profiles of the metabolites themselves (Figures 2G–K). The conversion ratio of IAA was significantly increased in NMOSD patients at the remission phase (85.8% ± 67.2%) when compared to the HC group (58.1% ± 29.5% and *p* = 0.04) (Figure 2J). Our data suggest that some of the Trp metabolites may be involved in the pathogenesis or endogenous repair initiation of NMOSD.

### Aromatic amino acids and their metabolites in CSF

**FIGURE 3:**
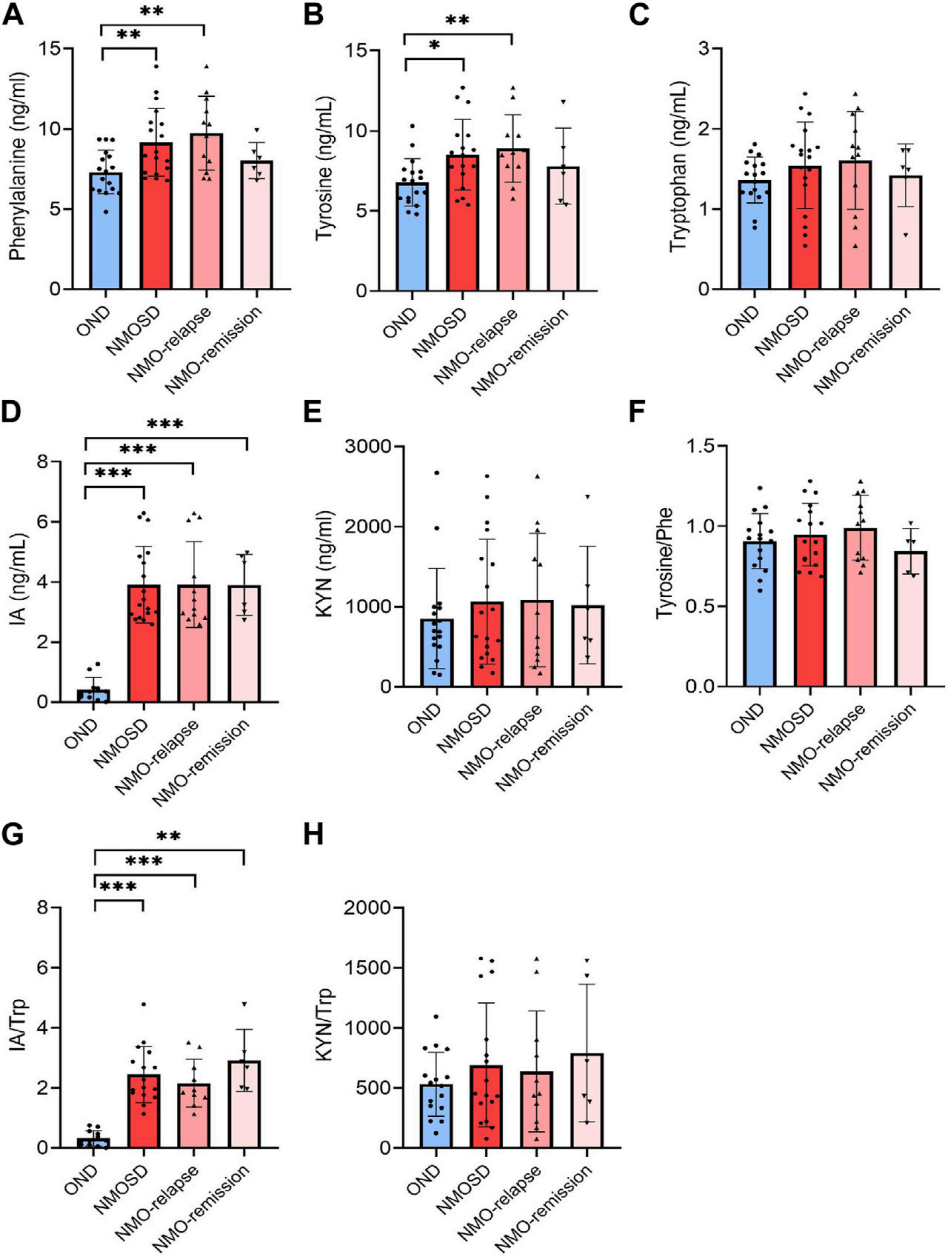
Levels of aromatic amino acids and their metabolites in cerebrospinal fluid (CSF) of neuromyelitis optica spectrum disorder (NMOSD) and other neurological disease (OND) patients. CSF samples were collected from 18 (12 in relapse and 6 in remission) NMOSD and 17 OND patients. Phenylalanine [Phe, **(A)**], tyrosine **(B)**, tryptophan [Trp, **(C)**] and metabolites 3-indoleacrylic acid [IA, **(D)**] and kynurenine [KYN) **(E)**] were analyzed using the liquid chromatography–tandem mass spectrometry (LC-MS/MS)-based method. The conversion profiles of tyrosine/Phe **(F)**, IA/Trp **(G)**, and KYN/Trp **(H)** are shown. Statistical significance was determined by the Student’s t-test or Mann–Whitney U test; data are presented as the mean ± SD. **p < 0.01 and ***p < 0.001.

A total of 12 aromatic amino acids and metabolite biomarkers were measured in the CSF derived from NMOSD and OND patients. However, due to the sensitivity and limitation of technique, the CSF levels of PAGln, HA, KYNA, HIAA, IAA, I-3-CA, and QUIN were below detectable values. Among all the detectable aromatic amino acids and metabolites, the CSF levels of Phe and tyrosine showed a significant increase in NMOSD patients (9.2 ± 2.2 ng/mL and 9.0 ± 3.0 ng/mL for Phe and tyrosine, respectively, *p* < 0.01 and *p* < 0.05), especially at the relapse stage (9.7 ± 2.3 ng/mL for Phe and 9.6 ± 3.3 ng/mL for tyrosine, respectively, *p* < 0.01, Figures 3A,B). The CSF levels of IA were markedly increased in NMOSD patients (3.9 ± 1.3 ng/mL) when compared to the OND group (0.4 ± 0.4 ng/mL, *p* < 0.001) and also increased obviously during both relapse (3.9 ± 1.4 ng/mL) and remission (3.9 ± 1.0 ng/mL) phases (Figure 3D). No significant difference was found in CSF Trp and KYN levels between NMOSD and OND patients (Figures 3C,E). There were no other significant differences observed in all detected amino acids and metabolites between the two NMOSD subgroups. The conversion profiles of Trp metabolite IA/Trp showed the same tendency as the level alteration profiles of the metabolites themselves (Figure 3G). No significant differences were found in the conversion ratio of tyrosine/Phe and KYN/Trp in the CSF samples among all detective groups (Figures 3F,H).

### Correlation of IA, HIAA, and I-3-CA levels in serum with clinical parameter EDSS

**FIGURE 4:**
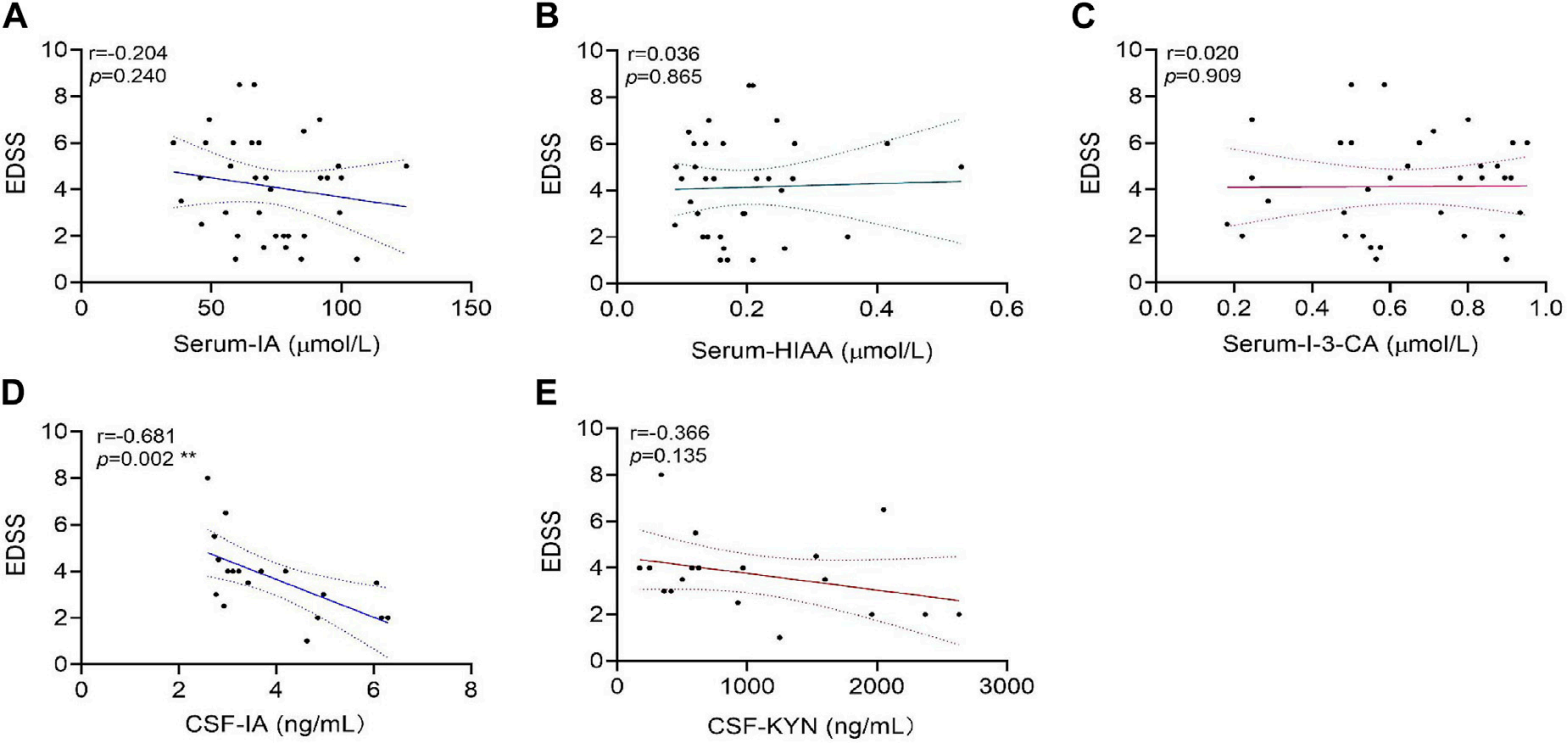
Correlations of tryptophan metabolite levels in serum or cerebrospinal fluid (CSF) samples with expanded disability status scale (EDSS) scores in neuromyelitis optica spectrum disorder (NMOSD) patients. The serum levels of tryptophan metabolites IA **(A)**, HIAA**(B)**, and I-3-CA **(C)** were analyzed for correlations with their EDSS scores in 47 NMOSD patients. The correlation of CSF levels for IA **(D)** and KYN **(E)** with EDSS scores in 18 NMOSD patients were analyzed. CSF-IA levels were correlated with the EDSS negatively (**p = 0.002, **(D)**. Correlation analyses were determined by Spearman’s rank correlation coefficient. Dashed lines refer to the standard errors.

In the NMOSD patients who had provided serum samples, the correlation analysis revealed that there were no significant associations between serum-IA levels and EDSS scores (r = −0.204 and *p =* 0.240), serum-HIAA and EDSS scores (r = 0.036 and *p =* 0.865), and serum-I-3-CA and EDSS scores (r = 0.020 and *p =* 0.909) (Figures 4A–C).

### Correlation of IA and KYN levels in CSF with clinical parameter EDSS

In the NMOSD patients who had provided CSF samples, correlation analyses revealed a significant negative correlation between CSF-IA and EDSS scores (r = −0.681 and *p =* 0.002) (Figure 4D). No significant correlations were found between CSF-KYN levels and EDSS scores (r = −0.366 and *p =* 0.135) (Figure 4E).

### Correlation of IA with GFAP and NfL in serum

**FIGURE 5:**
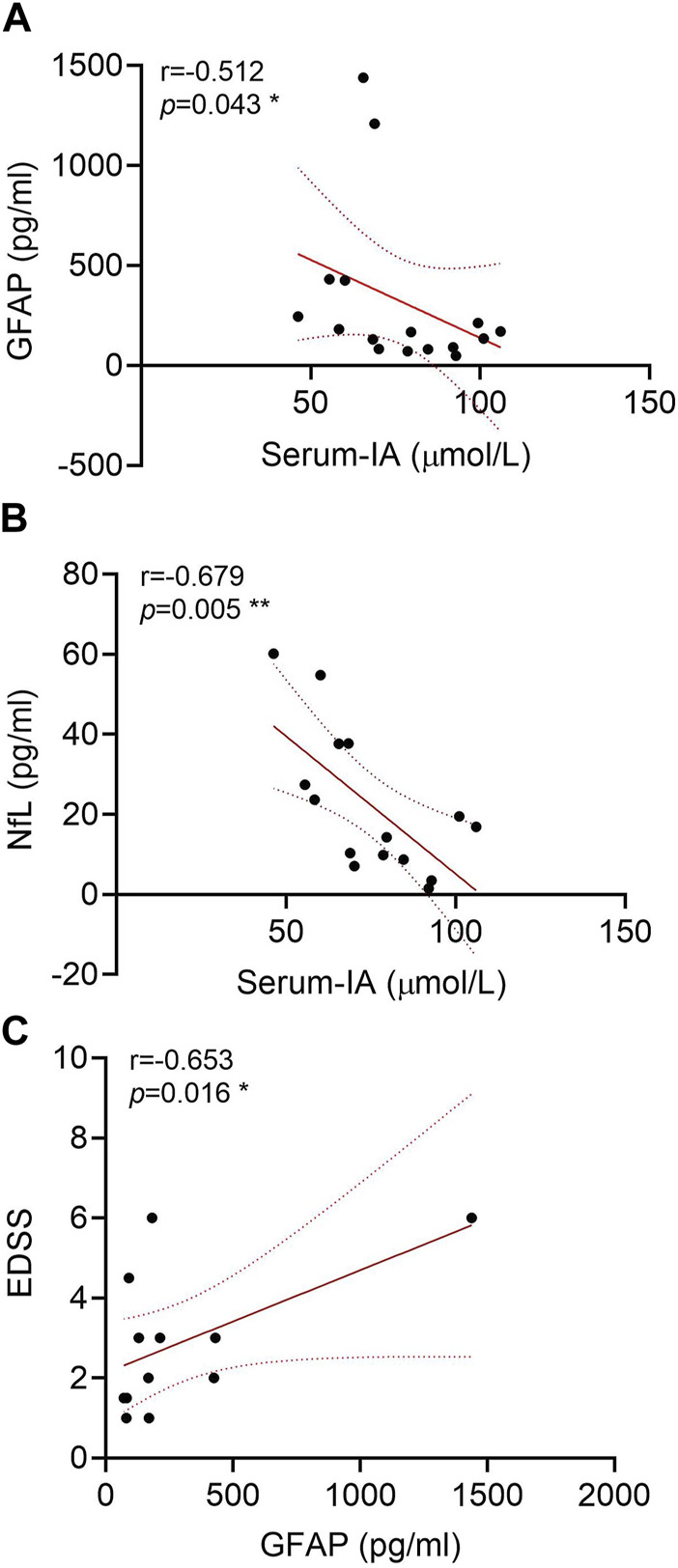
Correlations of glial fibrillary acidic protein (GFAP) and neurofilament light (NfL) in the serum of neuromyelitis optica spectrum disorder (NMOSD) patients with serum indoleacrylic acid (IA). Serum IA was negatively correlated with GFAP **(A)** and NfL **(B)** in NMOSD patients (n = 17). **(C)** Correlations of GFAP with the expanded disability status scale (EDSS) scores. Correlation analyses were determined by Spearman’s rank correlation coefficient. Dashed lines refer to the standard errors.

Based on the novel ultra-sensitive Simoa technology, the peripheral GFAP and NfL levels of 17 NMOSD patients who had provided serum samples were evaluated. The correlation analysis showed that the Trp metabolite IA was negatively correlated with GFAP (r = −0.512 and *p* = 0.043) (Figure 5A) and NfL (r = −0.679 and *p* = 0.005) levels in the serum (Figure 5B). A positive correlation between the GFAP and EDSS scores was observed markedly (r = 0.653 and *p* = 0.016) (Figure 5C), which is consistent with previous literature reports (Schindler et al., 2021).

### Anti-inflammatory effect of IA in *in vitro* astrocyte model injured by NMO-IgG

**FIGURE 6:**
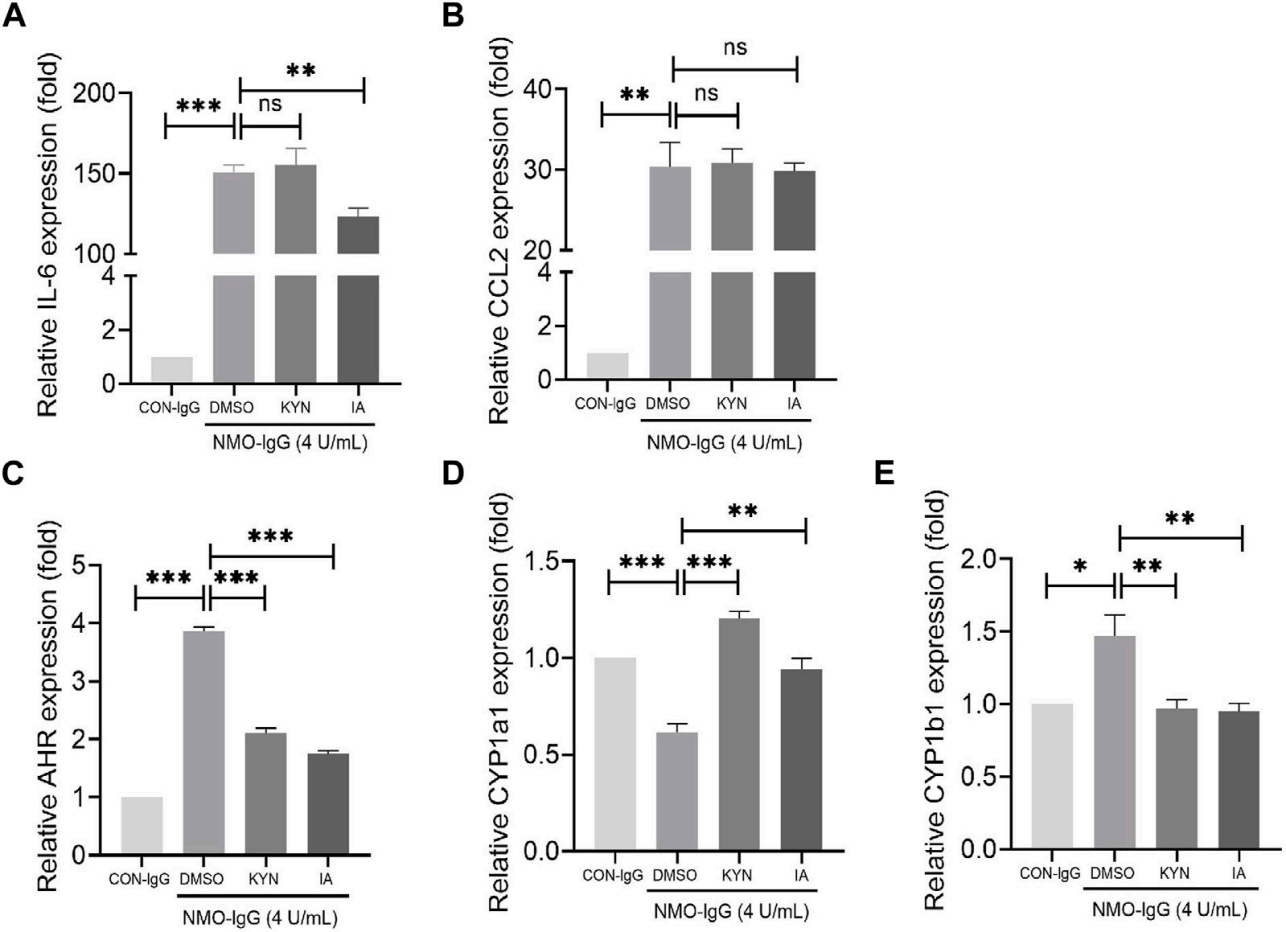
Effect of indoleacrylic acid (IA) on mRNA expressions of IL-6, CCL2, and nuclear transcription factor aryl hydrocarbon receptor (AHR) in an *in vitro* neuromyelitis optica spectrum disorder (NMOSD) model. Human-IgGs from NMOSD patients exposed to astrocytes represented one of the important events in NMOSD pathogenesis. Rat primary astrocytes were pre-incubated with IA (40 μM) or kynurenine (KYN, 50 μM, positive control) for 16 h and then stimulated with NMO-IgG (4 units/mL) for 4 h; mRNA levels were determined by RT-PCR. **(A)** IA preincubation decreased IL-6 mRNA expression significantly. **(B)** Changes in the CCL2 mRNA expression. **(C)** The AHR mRNA levels increased significantly by NMO-IgG stimulation, while both IA and KYN preincubation could reduce the AHR increase caused by NMO-IgG, and the inhibition efficiency was more potent in the IA group **(D, E)** Changes in the AHR downstream effector genes CYP1a1 and CYP1b1 expressions. Dimethyl sulfoxide (DMSO, 0.1%) was added as the vehicle control. Data are presented as the mean ± SD from at least three independent experiments. *p < 0.05, **p < 0.01, and ***p < 0.001, ns, no significance.

Stimulation of rat primary astrocytes with NMO-IgG (normalized by 4 units/mL AQP4-IgG) for 4 h resulted in the significant enhancement of IL-6 and CCL2 mRNA expressions (Figures 6A,B). When astrocytes were pre-treated with IA (40 µM) for 16 h (Du L. S. et al., 2020), the expressions of IL-6 mRNA decreased markedly. IA has a more potent effect on IL-6 increase than does KYN (Figure 6A), which is a known AHR agonist, suggesting that IA may be a more efficient and stronger anti-inflammatory ligand. Meanwhile, the expression of AHR following IA or KYN pre-treatment was significantly lower than that for the NMO-IgG stimulated group and still higher than that for the CON-IgG group (Figure 6C), indicating that AHR may mediate anti-inflammatory effects when the AHR ligand is present.

Upon AHR activation by its ligand, AHR translocates into the nucleus and dimerizes with the AHR nuclear translocator (ARNT); the heterodimer then binds to dioxin-responsive elements (DREs) located upstream of its target genes such as *CYP1a1* and *CYP1b1*, leading to a wide variety of toxic responses. IA or KYN pre-treatment increased the mRNA expressions of *CYP1a1* (Figure 6D) and decreased that of *CYP1b1* (Figure 6E) genes. The change trend of *CYP1b1* is consistent with that of AHR, indicating that *CYP1b1* may be the target gene located downstream that is activated by ligands in combination with the heterodimers in NMO-IgG damaged astrocytes.

## Discussion

We have shown in this study that some aromatic amino acids or their metabolites in the serum or CSF of NMOSD patients may wave and reflect disease activity and severity immediately. In the present study, we identified three aromatic amino acids and nine important metabolites, and the main findings are as follows: 1) tyrosine and Trp metabolite IA decreased significantly in the serum of NMOSD patients during both the relapse and remission phases; I-3-CA levels were remarkably lower in both NMOSD subgroups than in the OND group; HIAA was significantly increased during relapse and remission in NMOSD patients when compared to HC and OND groups; 2) the CSF levels of Phe and tyrosine showed a significant increase in NMOSD during relapse when compared to OND patients; Trp metabolite IA increased markedly in the CSF of NMOSD patients during both the relapse and remission phases when compared to the OND group, and the increased IA in the CSF showed a significant correlation with EDSS decline; 3) IA negatively correlated with peripheral GFAP and NfL levels in the serum; 4) as an AHR ligand, IA showed anti-inflammatory effects by alleviating IL-6 mRNA increase induced by NMO-IgG stimulation.

KYN, KYNA, and QUIN are all metabolites derived from the kynurenine pathway of Trp. Metabolites of the kynurenine pathway have both neurotoxic and neuroprotective effects. Disturbances in the kynurenine metabolic pathway in both the gut and brain may be associated with the pathogenesis of neuropsychiatric disorders (Pukoli et al., 2021). Accumulation of KYN in the brain has been associated with depression and schizophrenia (Metidji et al., 2018). The level of KYN in the serum is undetectable, and there is no significant difference in the level of CSF-KYN between the NMOSD and OND groups. Consistent with the aforementioned information, most patients who accepted the Hamilton Anxiety (HAMA) and Hamilton Depression (HAMD) scale evaluations show only mild or moderate anxiety or depression symptoms (Supplementary Table S1). No significant associations were found between the CSF KYN levels and EDSS scores. KYNA, a natural antagonist of the N-methyl-d-aspartate (NMDA) receptor, and QUIN, an agonist of the NMDA receptor, are considered to be endogenous excitotoxins in the brain. However, we did not obtain the KYNA and QUIN concentration profiles among all groups in this study. The possible physiological reasons for the serum levels of KYNA, KYN, and QUIN being below the detectable thresholds are as follows: KYN production derived from gut microbiota permeated through the blood–brain barrier and converted into neurotoxic metabolites such as 3-HK, QUIN, and KYNA, which induced further neuronal damage (Eryavuz et al., 2022). QUIN is produced from activated macrophages or microglia in the CNS. Recent studies suggest that the activation of macrophages with either interferon-gamma (IFN-γ), tumor necrosis factor-alpha (TNF-α), or other cytokines can lead to QUIN production (Pemberton et al., 1997). Elevated QUIN levels in the CNS showed a relationship with the severity of neurologic impairments and macrophage activation (Guillemin et al., 2003). The given QUIN is derived mainly from the microglia or macrophages, and the level of QUIN in the peripheral blood is lower. A study reported that mental symptoms may be related to QUIN levels (Kanchanatawan et al., 2018). However, no significant mental abnormalities were observed in the NMOSD patients. Patients who accepted the Anxiety and Depression scale evaluations showed only little or mild anxiety or depression symptoms (Supplementary Table S1). It may be another reason for the lower level of QUIN. The conversion from KYN to KYNA was catalyzed via kynurenic aminotransferase (KAT), in which KYNA acted as a neuroprotective agent under physiological conditions. Decreases in KAT enzyme activities were correlated with lower levels of KYNA in the brain section of multiple sclerosis patients (Fathi et al., 2022). We deduced that it may be a similar situation in NMOSD patients. Taken together, KYN may enter the CNS and further turn into other metabolites such as picolinic acid (PA) or xanthurenic acid (XA), resulting in a lower level of KYN in the serum.

All three aromatic amino acids are substrates of human gut microbiota, which produce dozens of metabolites that accumulate in the bloodstream. These small molecules commonly reach concentrations similar to those achieved by pharmaceutical agents, which are involved in diabetes (Dodd et al., 2017; Arneth et al., 2019), inflammatory bowel disease (Lavelle and Sokol, 2020), atherosclerotic cardiovascular disease (Xue et al., 2022), Alzheimer’s disease, and so on (Wu et al., 2021). Cognitive dysfunctions have been reported in 35%–67% of patients with NMOSD, specifically in attention, memory, and information processing speed (Eizaguirre et al., 2017). Tyrosine, an essential amino acid and the precursor of catecholamines, may improve the cognition and emotional state in patients by elevating brain catecholamines (Fazio et al., 2015; Mccann et al., 2021). We observed that tyrosine levels significantly decreased in the serum and increased in the CSF during relapse in NMOSD patients, suggesting that tyrosine concentration fluctuations may be associated with cognitive decline in patients, and the shortened reaction time of tyrosine imbalance may be associated with endogenous repair response activation and better cognitive performance during remission.

IA, HIAA, and I-3-CA are all metabolites of the essential amino acid Trp via the indole pathway, with rare reports of HIAA and I-3-CA functions. IA, produced by *Peptostreptococcus* species, has been reported to promote anti-inflammatory responses and may have therapeutic benefits in inflammatory bowel disease (Wlodarska et al., 2017). To our knowledge, metabolic characteristics of Trp metabolites in NMOSD patients have never been reported. In the present study, IA decreased in the peripheral (serum) and increased in the central (CSF) system of NMOSD patients during the acute phase, and we deduced that IA may penetrate into the CNS and have an impact on the mitigation of the immune inflammatory response in the brain. To evaluate the effect of IA on the inflammatory transcriptional response induced by NMOSD damage, we stimulated astrocytes with NMO-IgG, which enhanced IL-6 and CCL2 mRNA expressions. The mRNA level of AHR, a cytoplasmic receptor of IA, was also increased obviously by NMO-IgG stimulation. IA pre-treatment prevented IL-6 and AHR from increasing, suggesting that the AHR ligand IA may have an important anti-inflammatory function in NMOSD pathogenesis. However, there were no significant correlations between AQP4 antibody titers and IA levels in the serum (Supplementary Figure S3). During the relapse phase of NMOSD, IA entered into the CNS to mitigate the inflammatory response. It may be the reason why IA decreased in the periphery and increased in the CSF. Furthermore, IA had a weak effect on CYP1a1 but a potent effect on CYP1b1, suggesting that the anti-inflammatory effect of IA may mediate through targeting CYP1b1 transcription.

We report in this study a rapid and sensitive LC-MS/MS method for determining three aromatic amino acids and nine of their metabolites in a single run and within a few minutes. Despite the hydrophilic nature of some metabolites such as KYNA and QUIN, they cannot be detected in either the serum or CSF under present conditions. Metabolites that are not detected may be because their sample signals in the serum or CSF are below lower limits of quantitation (LLOQ). Of course, it would be fantastic if our approach could be further refined in the future to identify these metabolites. Furthermore, the method overcomes challenges of marked differences in endogenous baseline levels among analytes and has been applied over a wide concentration range (∼1,000 folds), not just for the serum in the periphery but is also sensitive enough to reliably measure most of the metabolites in the CSF. Recently, Simoa is an important technique in preclinical and clinical sample analyses, which can be easily applied and deliver robust results once established. NfL is a component of the neuronal cytoskeleton and is released into the CSF and blood after neuronal axonal injury, and it was used as a common biomarker for neuroaxonal damage (Chang et al., 2021). GFAP is a principal intermediate filament that contributes to the astrocytic cytoskeleton and represents a marker of astrocytic injury (Aktas et al., 2021; Chang et al., 2021). Simoa technology enables the detection of the two aforementioned markers in the serum. This method reveals that serum the NfL and GFAP levels are high in patients with NMOSD, and NfL and GFAP are regarded as biomarkers for neurons or astrocyte injury (Watanabe et al., 2019). However, the measurement of multiple compounds or several biomarkers together becomes increasingly important during different disease statuses, and LC-MS/MS enables multiple biomarker measurements simultaneously in a single study (Fuertig et al., 2016; Ma et al., 2017). The short analytical runtime, simple sample preparation procedure, and low sample volumes of only a few microliters of the serum and CSF can be reliably measured with adequate sensitivity. Approaches with more time- and cost-efficient analyses can be expanded to further molecules and research compounds. Targeted metabolomics techniques will increase our understanding of the pathophysiological process during disease development.

A limitation of our clinical sample study is its retrospective design without clinical follow-up information. The patient cohort was small, and not all NMOSD patients had their serum GFAP and NfL levels evaluated, or their anxiety and depression symptoms measured. We could not adequately analyze the levels of aromatic amino acids or their metabolites after the initial onset. Therefore, a well-organized longitudinal prospective study should be conducted in the future to confirm our findings. However, we believe that our data provide the foundation for future longitudinal studies that have to enroll more patients to establish the prognostic value and disease-activity monitoring potential of aromatic amino acids and their metabolites in NMOSD.

In conclusion, these findings underscore the potential key role of aromatic amino acid metabolites, especially IA, in the pathogenesis of NMOSD. During the relapse phase of NMOSD, IA decreased markedly in the serum and increased significantly in the CSF measured using the LC-MS/MS-based analytical method. Our *in vitro* data suggest that Trp metabolite IA can effectively inhibit the release of pro-inflammatory factors IL-6 from astrocytes induced by NMO-IgG, and its anti-inflammatory effect may be mediated by AHR, at least partially. This may indicate that IA is a promising biomarker candidate for disease activity and severity monitoring and improving symptoms of NMOSD, which is worth further exploration.

## Data Availability

The original contributions presented in the study are included in the article/Supplementary Materials; further inquiries can be directed to the corresponding authors.

## Supplementary material

SUPPLEMENTARY FIGURE S1 Serum metabolite levels in NMOSD patients who obtained immunosuppressive agent or not.

SUPPLEMENTARY FIGURE S2 Cerebrospinal fluid (CSF) metabolite levels in NMOSD patients who obtained immunosuppressive agent or not.

SUPPLEMENTARY TABLE 1 Anxiety and depression characterics evaluation in NMOSD patients who provided serum samples.
