# Supplementary material for: Profile and potential role of novel metabolite biomarkers, especially indoleacrylic acid, in pathogenesis of neuromyelitis optica spectrum disorders
Source: Front Pharmacol. 2023 May 19;14:1166085. doi: 10.3389/fphar.2023.1166085 (PMC10263123; doi:10.3389/fphar.2023.1166085)
Supplement: Supplementary file 1 [file Table1.DOCX]

**Methods**

***Anxiety or depression symptoms evaluation in some enrolled NMOSD patients who provided serum samples***

Hamilton Anxiety (HAMA) and Hamilton Depression (HAMD) scale at the time-point of sample collection were used to assess the anxiety and depression symptoms of NMOSD patients, respectively (Zimmerman *et al.*, 2013, Thompson, 2015). Symptoms reflection of psychic anxiety and somatic anxiety were all recorded according to the HAMA scale. Anxiety/somatization, weight, cognitive impairment, diurnal variation, blockage, sleep disorders, and hopelessness were all noted for the HAMA scale evaluation. It consisted of 2 subscales: scores below 7 referred to no symptom or mild symptom; scores between 7 to 14 referred to moderate symptoms. Scale evaluation was assessed by 2 fixed operators who did not participate in the metabolite measurement. To uniform evaluation terms and standardize the evaluation process, the operators received at least 1-week of scale evaluation training by experts before evaluation.

**Results**

***Anxiety or depression characterics in NMOSD patients***

Since tryptophan metabolites play crucial roles in physiological functions, and their imbalances are implicated in the pathology of anxiety or depression (Deng *et al.*, 2021), the symptom scores in anxiety and depression of some NMOSD patients who provide serum samples were evaluated. As shown in Supplementary Table 1, a total of 32 patients accepted the assessment, 6 in relapse and 26 in remission. Among the 6 patients during the relapse stage, four patients with HAMA scores below 7, and two patients with HAMA scores were in the range of 7 to 14. Interestingly, HAMD scores show similar profiles. The scores mean level of NMOSD patients during the relapse stage with anxiety and depression symptoms based on HAMA and HAMD scales were 3.3±4.1 (0-9) and 5.2±4.9 (0-13), respectively.

In 26 NMOSD patients during remission, 20 patients with HAMA scores below 7 and 6 patients with HAMA scores in the range of 7 to 14. HAMD scores for 23 patients below 7, and the HAMD scores of 3 patients were in the range of 7 to 14. The scores mean level of NMOSD patients during the remission stage with anxiety or depression symptoms based on HAMA and HAMD scales were 2.4±3.3 (0-12) and 3.4±3.9 (0-14), respectively. There were no significant differences in HAMA or HAMD scores between NMOSD relapse and remission subgroups (3.3±4.1 and 2.4±3.3 for HAMA, 5.2±4.9 and 3.4±3.9 for HAMD, respectively, *p*=0.613, *p*=0.351).

**References:**

Deng, Y., Zhou, M., Wang, J., Yao, J., Yu, J. & Liu, W., et al. (2021). Involvement of the microbiota-gut-brain axis in chronic restraint stress: disturbances of the kynurenine metabolic pathway in both the gut and brain. *Gut Microbes*. 13, 1, 1-16. doi: 10.1080/19490976.2020.1869501

Thompson, E. (2015). Hamilton Rating Scale for Anxiety (HAM-A). *Occup Med (Lond)*. 65, 7, 601. doi: 10.1093/occmed/kqv054

Zimmerman, M., Martinez, J. H., Young, D., Chelminski, I. & Dalrymple, K. (2013). Severity classification on the Hamilton Depression Rating Scale. *J Affect Disord*. 150, 2, 384-8. doi: 10.1016/j.jad.2013.04.028

Supplementary Table 1

Anxiety and depression characterics evaluation in NMOSD patients who provided serum samples.

|  | Scores | Relapse (n=6/12) | |  | Remission (n=26/35) | |
| --- | --- | --- | --- | --- | --- | --- |
|  |  | Anxiety  (HAMA) | Depression  (HAMD) |  | Anxiety  (HAMA) | Depression  (HAMD) |
|  | ＜7 | 4/6 | 4/6 |  | 20/26 | 23/26 |
|  | 7-14 | 2/6 | 2/6 |  | 6/26 | 3/26 |
|  | Mean ± SD  (range) | 3.3±4.1  (0-9) | 5.2±4.9  (0-13) |  | 2.4±3.3  (0-12) | 3.4±3.9  (0-14) |

Data were shown as means ± standard deviation (S.D.). Scores below 7 referred to no or mild symptoms; scores between 7 and 14 referred to moderate symptoms.

Supplementary Figure 1


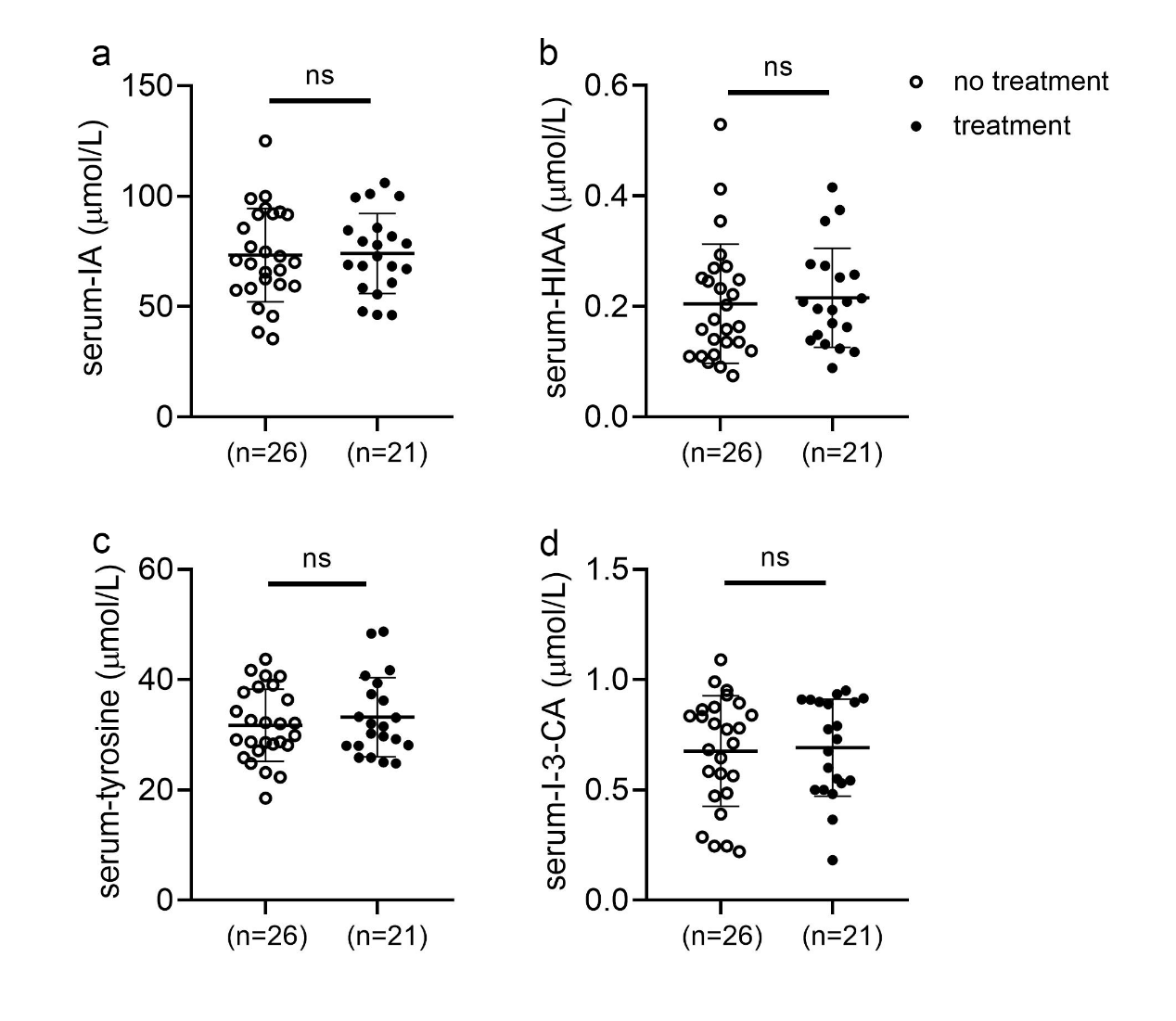


Serum metabolite levels in NMOSD patients who obtained immunosuppressive agent or not. Immunosuppressive drug treatments had no significant effect on serum levels of IA (a), HIAA (b), tyrosine (c) and I-3-CA (d) compared to NMOSD patients who were not treated. No treatment: the NMOSD patients did not administer immunosuppressive drugs within 6 months before blood sample collection; treatment: the NMOSD patient administered immunosuppressive drugs within 6 months. Statistical significance was determined by the student’s *t* test or Mann-Whitney U test. Data were presented as the mean ± SD. ns, no significance.

Supplementary Figure 2


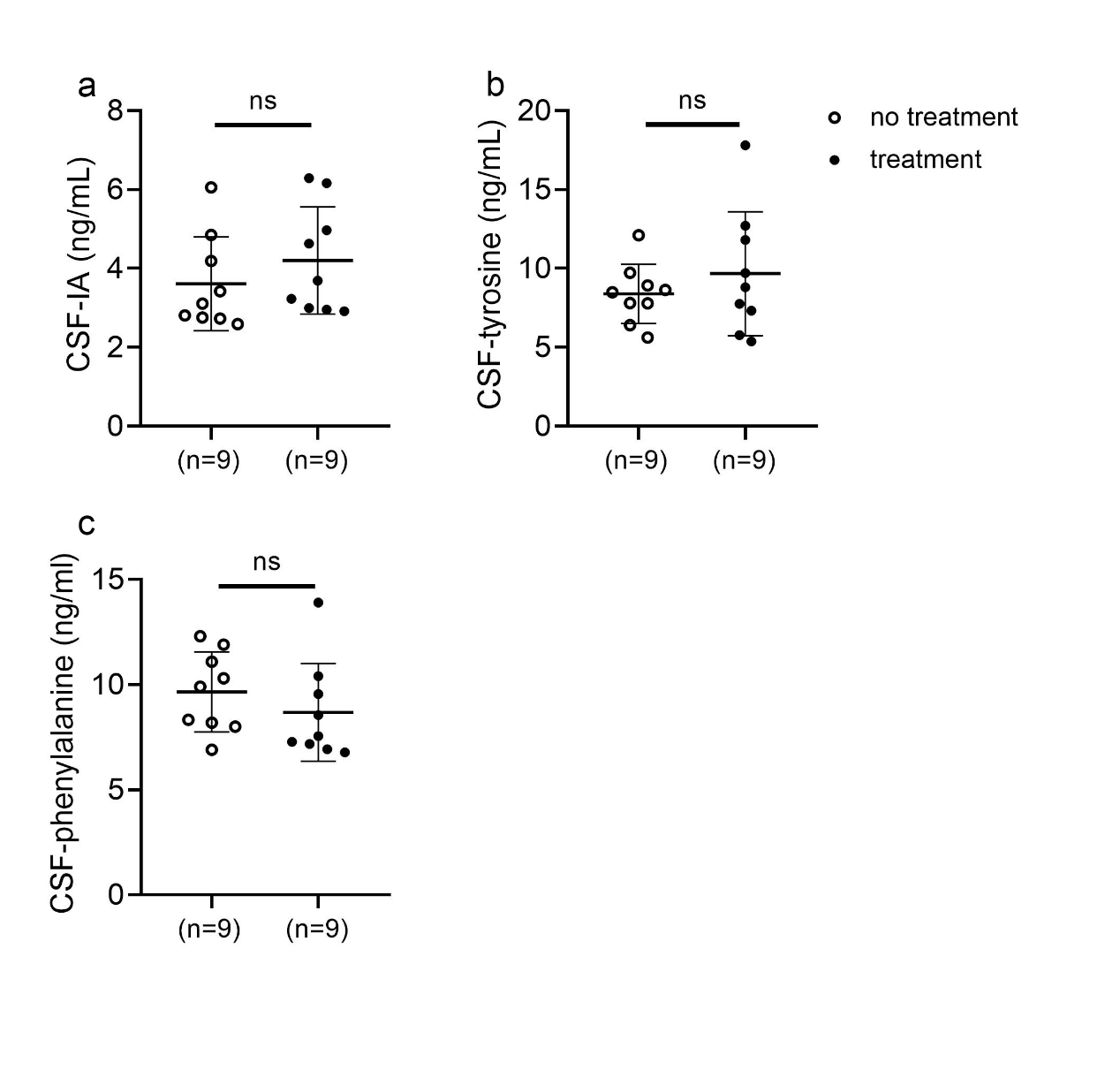


Cerebrospinal fluid (CSF) metabolite levels in NMOSD patients who obtained immunosuppressive agent or not. Immunosuppressive drug treatments had no significant effect on CSF levels of IA (a), tyrosine (b) and phenylalanine (c) compared to NMOSD patients without treatments. No treatment, the NMOSD patient did not treated with any immunosuppressive drugs within 6 months before CSF sample collection; treatment, the NMOSD patient were treated with immunosuppressive drugs within 6 months. Statistical significance was determined by the student’s *t* test or Mann-Whitney U test. Data were presented as the mean ± SD. ns, no significance.

Supplementary Figure 3


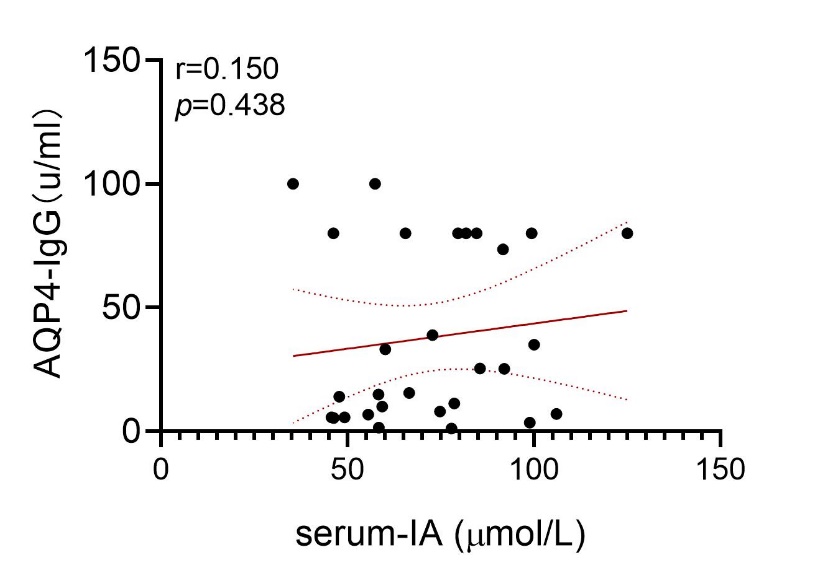


Correlations of AQP4 antibody titer and serum indoleacrylic acid (IA) levels in NMOSD patients. Correlation analyses were determined by Spearman's rank correlation coefficient. It was found that there was no significant correlation between serum AQP4 antibody titers and IA levels (*p*=0.438). The dashed lines refer to the standard errors.
